# Turnover intention and its associated factors among nurses: a multi-center cross-sectional study

**DOI:** 10.3389/fpubh.2023.1141441

**Published:** 2023-06-15

**Authors:** Yang Liu, Yinglong Duan, Meiying Guo

**Affiliations:** ^1^Nursing Department, The Third Xiangya Hospital, Central South University, Changsha, Hunan, China; ^2^Department of Emergency, The Third Xiangya Hospital, Central South University, Changsha, Hunan, China

**Keywords:** nursing, turnover intention, job satisfaction, pay level satisfaction, interpersonal conflict, factor, sense of belonging to hospital

## Abstract

**Aim:**

The purpose of our study was to assess the turnover intention of nurses in China and examine the associated factors.

**Background:**

Since the world population ages, the demand for nurses has kept growing, and the shortage of nurses and high turnover rates are concerned with the quality of care. Thus, understanding nurses' turnover intention and the relevant factors could provide nurse managers with strategies to address the modifiable factors to decrease the turnover rate of nurses.

**Methods:**

A multi-center cross-sectional study was conducted on a total of 1,854 nurses working in 15 hospitals in China. Data were collected using a self-designed demographic questionnaire, the Turnover Intention Scale, the Job Satisfaction Scale, the Pay Level Satisfaction Scale, the Interpersonal Conflict at Work Scale, and a single question on the sense of belonging to the hospital.

**Results:**

Most nurses (*n* = 1286, 69.4%) had a high level of turnover intention. Multilevel logistic regression analysis demonstrated that nurses being single (OR = 1.366, *p* < 0.05), with a junior college or below (OR = 0.381, *p* < 0.01), being a clinical nurse (OR = 1.913, *p* < 0.01), having higher pay level (OR = 0.596, *p* < 0.001), having higher job satisfaction (OR = 0.406, *p* < 0.001), having conflicts with colleagues (OR = 1.400, *p* < 0.05), and having a higher sense of belonging to the hospital (OR = 0.532, *p* < 0.001) proved to affect nurses' turnover intention.

**Conclusion:**

This study extended the knowledge about the factors associated with nurses' intention to leave, which led to the turnover of nurses, and is one of the main contributors to the current shortage of nurses.

**Implications for nursing management:**

This study provided new approaches to decreasing the turnover rate of nurses. Effective management strategies may mitigate nurses' turnover intention.

## 1. Introduction

An adequate number of human resources is the fundamental condition to ensure the quality and safety of nursing (1). The shortage of nurses is a critical issue that the healthcare system faces worldwide (2). Moreover, there is a growing demand for health services with the aging of the population. Despite the high demand for nurses, there is still a considerable proportion of nursing staff with turnover intention, which refers to their psychological tendency to leave their current work environment. It predicts actual turnover actions and it can be either voluntary or involuntary (3). A New Zealand study (4) found an average turnover rate per year of 44% among nurses. Similarly, a cross-sectional observational study (5) conducted in 10 European countries suggested that 33% of nurses intend to leave their employment at the time. Notably, nurses' turnover intention poses a severe threat to the quality of care and patient outcomes (6). Furthermore, the increasing proportion of turnover increases the workload of other nurses, which may lead to more nurses intending to leave as well. Therefore, it is critical to find the variables with turnover intention among nurses.

Job satisfaction is a complex and multifaceted concept that is subjectively dependent on job characteristics and personal expectations (7, 8). It can be measured in both general and specific ways, with the former identifying overall attitudes toward the job and the latter pinpointing specific aspects of job dissatisfaction (9). A cross-sectional survey (10) conducted in Malaysia found that job satisfaction was associated with their intention to leave their current employment, with those who have a high degree of job satisfaction having a higher retention rate (11). Job satisfaction can often drive a nurse's decision to stay or go (12). Payment, which refers to the appropriate reward that nurses receive for their effort, skills, and other personal input, is a crucial aspect of job satisfaction. The relationship between pay level satisfaction and turnover intentions among nurses may be more dependent on the balance between work and pay level (13), rather than the amount of money. Park et al. (14) found that pay level satisfaction was the most visible and long-lasting predictor of turnover intentions. Wang et al. (15) evaluated the turnover intentions of 538 nurses, and the results indicated that both payment and psychological reward probably affected the turnover intentions. Interpersonal conflict, which includes aggressive behavior such as spreading gossip or being rude to coworkers, is relatively common among nurses and may increase the risk of high turnover intentions generated from various sources, including coworkers, supervisors, and patients (16, 17). Coworkers' and supervisors' conflict is linked to both individual and organizational outcomes among nurses. It potentially causes increased absenteeism and even a desire to quit (18). Those who experience conflict reported high levels of turnover intentions. A sense of belonging, which refers to being socially connected and recognized as part of a group, is also vital for job satisfaction (19). Wei et al. (20) demonstrated that nurses would be more likely to stay when they feel a sense of belonging to their work environment, which helps to practice nursing more effectively.

While turnover intention among nurses has been measured in many countries, there is limited knowledge about its prevalence and associated factors in China. Therefore, studies with large sample sizes on nurses' intention to leave are warranted to provide clues for nurse managers to retain nursing staff. This study aimed to investigate the turnover intentions among nurses in China and explore the factors related to them.

The theoretical framework of this study was based on the Price–Mueller model (21) and was used to determine the factors influencing turnover intentions among Chinese nurses. Price et al. (21) synthesized the external factors that affect turnover and divided them into environmental, personal, and structural variables. Job satisfaction, pay level satisfaction, interpersonal conflict, and sense of belonging were reported as the main variables influencing turnover intentions in previous studies (10, 14) and were identified as structural and environmental variables in our study. Kim et al. (22) identified individual variables as the most influential among other relevant variables on turnover intention, such as age, marital status, and education level. Thus, the above sociodemographic characteristics were also considered as individual variables in this study. The hypothesis is that low levels of job satisfaction, pay level satisfaction, sense of belonging, and high levels of interpersonal conflict were related to higher turnover intentions among nurses.

## 2. Methods

### 2.1. Study design

This study was a cross-sectional descriptive survey to explore the effects of job satisfaction, pay level satisfaction, interpersonal conflict at work, and sense of belonging to the hospital on nurses' intention to leave. It was reported following by the STROBE Statement—a checklist of items that should be included in reports of observational studies. The ethics approval was obtained from the study hospital.

### 2.2. Participants and settings

The participants were recruited from 15 hospitals in the Hunan province of China between December 2021 and February 2022. The inclusion criteria were registered nurses who were 1) engaged in clinical nursing work in the hospital and 2) gave informed consent to participate in the research. The nurses who had major physical illnesses, such as malignant tumors, and acute disease conditions during the investigation period were excluded.

### 2.3. Measurements

#### 2.3.1. Sociodemographic characteristics

The sociodemographic characteristics were collected by a self-designed questionnaire that included age, gender, single or not, education level, length of employment, technical titles, post, emergency, and critical care department or not.

#### 2.3.2. Turnover intention scale

The turnover intention scale was compiled by Michaels and Spector in 1982 (23). Li Dongrong and Li Jingyuan translated and revised it for Chinese employees. The Chinese version has six items in total. Item 1 and item 6 measure the possibility of employees quitting their current job; item 2 and item 3 reflect employees' motivation to find other jobs, and item 4 and item 5 indicate the possibility of an employee getting an external job. The scale adopts Likert's 4-level score. Response options ranged from 1 (never) to 4 (often). The total score of the scale is the average score of each item. The higher the score, the stronger the turnover intention is. The total average score of turnover intention ≤ 2 indicates a low turnover intention, and the total average score of turnover intention > 2 indicates a high turnover intention. The Chinese version of the scale had a Cronbach's α of 0.773 (24). In our study, Cronbach's α coefficient of the scale is 0.824.

#### 2.3.3. Job satisfaction scale

Job satisfaction was measured with the 3-item overall job satisfaction scale and it can be used for Chinese employees (25). The three questions were “Generally speaking, I don't like my work”, “Generally speaking, I am satisfied with my work”, and “Generally speaking, I like working here”. Response options ranged from 1 (Strongly disagree) to 6 (Strongly agree). The higher the score of job satisfaction, the more satisfied the job is. In our study, Cronbach's α coefficient of the scale is 0.754.

#### 2.3.4. Pay level satisfaction scale

The pay level satisfaction scale was developed by Tang et al. (26). The scale consists of four items. Response options ranged from 1 (strongly dissatisfied) to 5 (strongly satisfied). The higher the score of pay level satisfaction, the more satisfied the pay level is. The Cronbach's α coefficient of the scale is 0.9 (26). In our study, Cronbach's α coefficient of the scale is 0.958.

#### 2.3.5. Interpersonal conflict at work scale

The interpersonal conflict at work scale was developed to reflect interpersonal conflict at work and consisted of eight items, including two dimensions: conflict with the supervisor and conflict with coworkers (27). Each item used a 5-point response scale that ranged from never (1) to very often (5). Cronbach's alpha coefficients for conflict with supervisors and conflict with coworkers are 0.86 and 0.85. In our study, Cronbach's α coefficient of conflict with supervisors and conflict with coworkers are 0.931 and 0.945, respectively.

#### 2.3.6. Sense of belonging to hospital

The sense of belonging to the hospital was assessed using one single question. In a previous study, a single question item was used to evaluate the sense of community belonging: “How would you describe your sense of belonging to your local community? (28), and was widely used in other studies (29–31). In this study, we changed the ‘local community' to ‘hospital' to indicate the respondents' sense of belonging in their hospitals. The responses are: very strong (4), somewhat strong (3), somewhat weak (2), or very weak (1).

### 2.4. Data collection procedure

Data for this study were collected from 15 different hospitals in Hunan, China, between December 2021 and January 2022. The survey was distributed through an online platform (https://www.wjx.cn/), with eligible participants receiving a link to the questionnaires. At the start of the survey, participants were provided with detailed information about the research, including its purpose, potential benefits, and harms. They were then asked to provide their informed consent to participate in the study and complete the questionnaires. It took approximately 15–20 min to complete the questionnaire, and participation was voluntary.

### 2.5. Data analysis

The SPSS version 26 statistical software was used for data analysis. Continuous variables were presented as mean ± standard deviation, whereas categorical variables were presented as number and percentage. Univariable analysis, including *t*-test and Chi-square test, was conducted to examine the differences between nurses with low or high turnover intention in terms of different sociodemographic characteristics, job satisfaction, pay level, interpersonal conflict, and sense of belonging to the hospital. Subsequently, a multilevel logistic regression model was used to identify potential factors that might affect the turnover intentions of nurses with turnover intention as the dependent variable and the factors with *p* < 0.05 in the univariable analysis as the independent variables. The *p*-value of < 0.05 indicated statistical significance.

## 3. Results

### 3.1. Sociodemographic characteristics and turn over intention

The social-demographic characteristics of the 1,854 participants are presented in Table 1. The mean age of the participants in the survey was around 31 years old (*SD* 13.32), with the majority being female (94.2%), not single (76.1%), and having had at least a bachelor's degree (70.8%). Regarding other work-related characteristics, most participants worked in the hospital for more than ten years (42.2%), held a primary title (56.8%), and did not work in emergency or critical care departments (69.5%). Additionally, more than 90% of participants were clinical nurses, and 69.4% (*n* = 1286) had a high turnover intention (score> 2).

**Table 1:** Univariable analysis of turnover intention.

	**All participants (*n =* 1854)**	**low turnover intention (*n =* 568)**	**high turnover intention (*n =* 1286)**	***t/chi-square***	**p-value**
**Age**	31.37 ± 13.32	33.28 ± 19.20	30.53 ± 9.52	3.245^a^	0.001
**Gender**					
Male	108 (5.8%)	28 (4.9%)	80 (6.2%)	1.197	0.279
Female	1746 (94.2%)	540 (95.1%)	1206 (93.8%)		
**Single or not**					
Single	444 (23.9%)	112 (19.7%)	332 (25.8%)	8.044	0.005
No single	1410 (76.1%)	456 (80.3%)	954 (74.2%)		
**Education level**					
Junior college or below	463 (25.0%)	172 (30.3%)	291 (22.6%)	14.049	0.001
Bachelor	1313 (70.8%)	379 (66.7%)	934 (72.6%)		
Master degree and above	78 (4.2%)	17 (3.0%)	61 (4.7%)		
**Length of employment**					
≤ 3years	261 (14.1%	78 (13.7%	183 (14.2%)	9.188	0.027
3-5years	225 (12.1%)	59 (10.4%)	166 (12.9%)		
5-10years	586 (31.6%)	163 (28.7%)	423 (32.9%)		
>10years	782 (42.2%)	268 (47.2%)	514 (40.0%)		
**Tiles**					
Primary title	1053 (56.8%)	296 (52.1%)	757 (58.9%)	7.652	0.022
Intermediate title	742 (40.0%)	250 (44.0%)	492 (38.2%)		
Senior title	59 (3.2%)	22 (3.9%)	37 (2.9%)		
**Post**					
Clinical nurse	1727 (93.1%)	509 (89.6%)	1218 (94.7%)	16.057	< 0.0001
Nurse manager	127 (6.9%)	59 (10.4%)	68 (5.3%)		
**Whether working at emergency or critical care department**					
No	1289 (69.5%)	416 (73.2%)	873 (67.9%)	5.331	0.021
Yes	565 (30.5%)	152 (26.8%)	413 (32.1%)		
**Job satisfaction**	4.05 ± 0.85	4.62 ± 0.71	3.80 ± 0.79	21.371	< 0.0001
**Pay level satisfaction**	3.02 ± 0.81	3.48 ± 0.84	2.81 ± 0.70	16.650 ^a^	< 0.0001
**Conflict with supervisor**	1.34 ± 0.68	1.16 ± 0.54	1.42 ± 0.73	−8.302 ^a^	< 0.0001
**Conflict with co-workers**	1.29 ± 0.62	1.14 ± 0.48	1.35 ± 0.66	−7.498 ^a^	< 0.0001
**Sense of Belonging**	2.56 ± 0.70	3.02 ± 0.73	2.36 ± 0.58	20.645	< 0.0001

^A^variance uneven using t'-test.

### 3.2. Univariate analysis results

The results of the univariate analysis showed that the level of turnover intention among nurses differed significantly in terms of age, single or not, educational level, length of employment, titles, post, whether working in emergency or critical care department, job satisfaction, pay level satisfaction, conflict with supervisor or coworkers, and sense of belonging (*p* < 0.05) (Table 1).

### 3.3. Multiple logistic regression analysis

As shown in Table 2, the results revealed that single nurses were more inclined to leave their jobs than nurses who were not single, which was statistically significant (OR 1.366, *p* < 0.05). Compared to nurses with a master's degree and above, nurses with a junior college degree or below were found significantly to have a smaller intention to leave (OR 0.381, *p* < 0.01). Apart from that, clinical nurses were more likely to intend to leave than nurse managers (OR 1.913, *p* < 0.01). The higher the job satisfaction and pay level of nurses, the lower intention to leave (OR 0.41, *p* < 0.001; OR 0.60, *p* < 0.001). The results also indicated that nurses who had conflicts with coworkers were more likely to have a tendency to leave (OR 1.400, *p* < 0.05). In other words, an increase of one unit in conflicts with coworkers increases the risk of turnover intention by OR 1.400. In contrast, the stronger the sense of belonging to the hospital, the less likely it is to tend to leave (OR 0.532, *p* < 0.001), that is, an increase of one unit in the sense of belonging to the hospital decreases the risk of turnover intention by OR 0.532.

**Table 2:** Multilevel logistic regression analysis of the relationship between individual characteristics, job satisfaction, pay level satisfaction, conflict with a supervisor, conflict with co-workers, sense of belonging, and turnover intention.

	**Turnover intention (N = 568/1286) Odds Ratio [95% CI]**	
	**Model 1**	**Model 2**
Age	0.975 [0.949–1.002]	0.988 [0.974–1.003]
male	1.084 [0.683–1.721]	1.035 [0.595–1.800]
Female	1	
Single	1.366^*^ [1.030–1.811]	1.086 [0.773–1.526]
No single	1	
Junior college or below	0.381^**^ [0.211–0.689]	0.327^**^ [0.164–0.651]
Bachelor	0.580 [0.330–1.019]	0.504^*^ [0.263–0.965]
Master degree and above	1	
≤ 3years	0.649 [0.385–1.095]	1.057 [0.610–1.832]
3–5years	0.863 [0.529–1.408]	1.013 [0.591–1.738]
5–10years	0.904 [0.637–1.284]	0.935 [0.641–1.365]
>10years	1	
Primary title	0.835 [0.409–1.704]	0.639 [0.290–1.411]
Intermediate title	0.701 [0.377–1.302]	0.497 [0.246–1.002]
Senior title	1	
Clinical nurse	1.913^**^ [1.254–2.92]	1.128 [0.704–1.805]
Nurse manager	1	
Not work at emergency or critical care department	0.839 [0.667–1.054]	1.144 [0.876–1.494]
Work at emergency or critical care department	1	1
Job satisfaction		0.406^***^ [0.330–0.499]
Pay level satisfaction		0.596^***^ [0.495–0.718]
conflict with supervisor		1.111 [0.826–1.495]
conflict with co-workers		1.400^*^ [1.010–1.940]
Sense of Belonging		0.532^***^ [0.431–0.657]
−2 log likelihood	2225.183	1718.989
Cox and Snell R^2^	0.032	0.263
Nagelkerke R^2^	0.045	0.371

Model 1 is adjusted for age, gender (male or female), single or not (single or no-single), education level (junior college or below, bachelor, master degree and above), length of employment ( ≤ 3 years, 3-5 years, 5-10 years, and >10 years), technical titles (primary title, intermediate title, and senior title), post (clinical nurse and nurse manager), work at emergency or critical care department (no, yes). Model 2 is adjusted for variables in model 1: job satisfaction, pay level satisfaction, conflict with supervisor, conflict with coworkers, sense of belonging, and I ^*^p < 0.05; ^**^p < 0.01; ^***^p < 0.001.

## 4. Discussion

Enhancing nurse retention rates is a crucial goal worldwide. This study aimed to evaluate the factors associated with nurses' intention to leave their jobs and provide valuable insights for nurse managers to retain their staff. Our findings revealed that a high percentage (69.4%, *n* = 1,286) of nurses expressed their intention to leave their current employment, indicating a need for serious consideration of turnover intentions in China. Furthermore, nurses with junior college or below, higher pay levels, job satisfaction, and a sense of belonging to the hospital were less likely to leave, whereas single nurses, clinical nurses, and those with conflicts with colleagues had a higher intention to leave.

In this study, nurses with a master's degree and above were more likely to leave their jobs than those with a junior college degree or below, which was in line with Ramoo et al. (10). This trend may be attributed to the difficulty nurses with higher education levels face in realizing their self-worth in a work environment where they perform the same duties as those with lower education levels. Therefore, nurse managers should focus on individual strengths and potentials to enhance nurses' sense of accomplishment and job satisfaction, especially for those with a master's degree or above, to increase their intention to stay in their jobs.

Moreover, our study found that single nurses had a stronger intention to quit than non-single nurses, which is consistent with that of Liang et al. (32) research in China. It may be explained that non-single nurses take on more family responsibilities, such as supporting their family financially, which may motivate them to stay in their jobs. In addition, the results suggested that clinical nurses were more likely to intend to leave their current employment compared to nurse managers, possibly because clinical nurses have to work shifts and handle more clinical workloads. Nurse managers could address this by designing career objectives based on individual nurses at different stages of their careers (33), providing more opportunities for career progression, thus increasing job satisfaction and reducing turnover intention.

The higher the pay level of nurses, the lower the level of intention to depart, which was in line with Wang et al. (15). As a facilitating factor, pay level motivates nurses to work and reduces their discontent and turnover intentions. It demonstrated that providing a more competitive salary and benefits package for staff can be a vital means of attracting and retaining talented professionals (14). There is an imbalance between effort and gains in the workplace, and the perception of this imbalance may be a critical factor in arousing nurses' turnover intentions. Hence, it is essential to focus on listening to nurses in formulating a scientific and reasonable salary policy to create a humanistic working environment, which will have a significant and lasting impact on turnover intention.

Additionally, the current study also revealed that nurses with higher job satisfaction were less likely to intend to leave. The result is supported by the outcome of the investigation in Korea reported by Choi et al. (34). This may be related to the work environment and job stress. When nurses are displeased with their jobs, the occurrence of turnover intentions is an important way for them to express their emotions. The excessive workload can cause exhaustion, leading to reduced job satisfaction among specialist nurses. Similarly, a supportive work environment may improve nurses' job satisfaction (35), and it is particularly relevant to develop concrete and varied measures to promote a better work environment. In addition, implementing a supportive leadership style may increase job satisfaction, deliver high-quality care, and lower nurse turnover intentions (36). Furthermore, workload allocation is necessary to ensure that nurses can balance their work and life.

What else was found is that conflicting with coworkers was associated with nurses' intention to leave. This finding was in line with the results reported by Lee et al. (37). When there is a conflict with coworkers, interpersonal relationships can be in a negative state (38). Interpersonal conflicts can create a negative work environment, leading to inefficiency, a stressful work environment, poor quality of care for patients, and low job satisfaction among nurses (16). Therefore, nursing managers should be equipped to manage conflicts among nurses, as it can harm healthy working relationships and lead to turnover. Efficient conflict management hinges on clear coordination and comprehension of disagreements. Nurses ought to be well educated about conflict-related knowledge and evidence-based strategies for conflict resolution to enable early resolution.

Regarding the sense of belonging to the hospital, our study has shown that the stronger the feeling, the lower is their intention to leave. It is in accordance with Reinhardt's findings (19), which suggested that a sense of belonging facilitates meaningful nursing practice and nurse career retention. A sense of belonging describes the loyalty and dedication of nurses to the hospital (39). Therefore, nurse managers should prioritize creating a healthy work environment to promote a sense of belonging among nurses. They should focus on the career development of nurses, maintain their organizational attitudes, and foster a positive work environment to enhance their sense of belonging.

## 5. Strengths and limitations

The strength of our research is that it is a multi-center study with a large sample size. The nurses surveyed were from 15 separate hospitals, and the results were highly credible. Nevertheless, it also has its weaknesses. First, the research design adopted was a cross-sectional study, which could not speculate on causality. Second, one item was used to measure the sense of belonging to the hospital. A sense of belonging is an abstract construct that is difficult to measure, and using a single item to measure it may not provide a complete assessment. Third, although it was a multi-center study, it was conducted in one province, which may have constrained the generalizability of the results. Future research could expand the geographic domain and utilize longitudinal studies to observe changes in nurses' intention to leave.

## 6. Conclusion

We found that most nurses had a high level of turnover intention. Nurses with junior college or below, higher pay level, higher job satisfaction, and a stronger sense of belonging to the hospital were less likely to consider leaving, while single nurses, clinical nurses, and having conflicts with colleagues were more likely to consider leaving. To reduce turnover intention, possible interventions include improving these associated factors identified in our study. Thus, it is crucial to focus on the psychological status of the imbalance between effort and income and establish a reasonable pay policy. Furthermore, creating a sound working atmosphere can also enhance the job satisfaction of nurses. Nurses' sense of professional identity and space for promotion also impact the sense of belonging to the hospital. The timely actions taken by nursing managers to prevent the emergence of turnover intentions are imperative. In addition, further studies are required to confirm the influential factors affecting nurses' intention to leave and to identify its changing trends.

## 7. Implications for nursing management

To address the shortage of nurse manpower, achieve the self-worth of nurses, and improve the quality of care (5), reducing the turnover rate of nurses is critical. Based on the results of our research, nurse managers could then focus on the modifiable factors associated with turnover intention, such as improving pay level, job satisfaction, and sense of belonging to the hospital to alleviate conflicts between colleagues. A favorable work environment that considers interpersonal conflict with colleagues is crucial to increasing nurses' job satisfaction. (7). Additionally, payment and psychological rewards (40) and appreciation of professional skills (41) might also increase nurses' loyalty and sense of belonging to the hospital, leading to higher retention rates. Therefore, nurse managers should enhance the equity of the compensation policies and encourage nurses to work toward the same goal.

## Data availability statement

The original contributions presented in the study are included in the article/supplementary material, further inquiries can be directed to the corresponding author.

## Ethics statement

The studies involving human participants were reviewed and approved by the Ethics Committee of The Third Xiangya Hospital of Central South University. The participants provided their written informed consent to participate in this study.

## Author contributions

YL wrote the initial draft of the manuscript. YD participated in the data processing and statistical analysis. MG participated in the designing of the study and in the administration of the questionnaire. All authors discussed the analytical results, and read and approved the final manuscript.
